# Brain functional connectivity and neuropsychiatric symptoms in patients with mild cognitive impairment, cerebrovascular disease and Parkinson disease

**DOI:** 10.1002/alz.087996

**Published:** 2025-01-03

**Authors:** Neda Rashidi‐Ranjbar, Nathan W Churchill, Sandra E Black, Sanjeev Kumar, Maria Carmela Tartaglia, Morris Freedman, Anthony E Lang, Thomas Steeves, Richard H Swartz, Gustavo Saposnik, Paula M McLaughlin, Sean Symons, Stephen C. Strother, Bruce G. Pollock, Tarek K. Rajji, Miracle Ozzoude, Brian Tan, Stephen R. Arnott, Robert Bartha, Michael Borrie, Mario Masellis, Stephen H Pasternak, Andrew R Frank, Dallas Seitz, Zahinoor Ismail, David F. Tang‐Wai, Leanne Casaubon, Jennifer Mandzia, Christopher J. M. Scott, Dar Dowlatshahi, David A. Grimes, Connie Marras, David G. Munoz, Joel Ramirez, Courtney Berezuk, Melissa F. Holmes, Corinne E. Fischer, Tom A. Schweizer

**Affiliations:** ^1^ Keenan Research Centre for Biomedical Science, Li Ka Shing Knowledge Institute, St. Michael’s Hospital, Toronto, ON Canada; ^2^ Keenan Research Centre for Biomedical Science, Li Ka Shing Knowledge Institute, St. Michael’s Hospital, Canada, TORONTO, ON Canada; ^3^ Heart and Stroke Foundation Canadian Partnership for Stroke Recovery, Toronto, ON Canada; ^4^ University of Toronto, Toronto, ON Canada; ^5^ Sandra Black Centre for Brain Resilience and Recovery, Sunnybrook Research Institute, Toronto, ON Canada; ^6^ Division of Neurology, Department of Medicine, University of Toronto, Toronto, ON Canada; ^7^ LC Campbell Cognitive Neurology Research Unit, Sunnybrook Research Institute, University of Toronto, Toronto, ON Canada; ^8^ Toronto Dementia Research Alliance, Toronto, ON Canada; ^9^ Temerty Faculty of Medicine, University of Toronto, Toronto, ON Canada; ^10^ Centre for Addiction and Mental Health, Toronto, ON Canada; ^11^ Institute of Medical Science, University of Toronto, Toronto, ON Canada; ^12^ Division of Neurology, Krembil Neuroscience Centre, Toronto Western Hospital, University Health Network Memory Clinic, Toronto, ON Canada; ^13^ Toronto Western Hospital, Tanz Centre for Research in Neurodegenerative Disease, Toronto, ON Canada; ^14^ Rossy PSP Program, University Health Network and the University of Toronto, Toronto, ON Canada; ^15^ Baycrest Health Sciences, Toronto, ON Canada; ^16^ Department of Medicine, University of Toronto, Toronto, ON Canada; ^17^ Rotman Research Institute, Baycrest Health Sciences, Toronto, ON Canada; ^18^ The Edmond J. Safra Program in Parkinson’s Disease and Morton and Gloria Shulman Movement Disorders Clinic, Toronto, ON Canada; ^19^ Movement Disorder Clinic, Toronto Western Hospital, University Health Network, Toronto, ON Canada; ^20^ Krembil Brain Institute, Toronto, ON Canada; ^21^ Tanz Centre for Research in Neurodegenerative Diseases, University of Toronto, Toronto, ON Canada; ^22^ Sunnybrook Health Sciences Centre, Toronto, ON Canada; ^23^ Li Ka Shing Knowledge Institute, Toronto, ON Canada; ^24^ Ontario Neurodegenerative Disease Research Initiative, Toronto, ON Canada; ^25^ Nova Scotia Health, Halifax, NS Canada; ^26^ Physical Sciences, Sunnybrook Research Institute, Toronto, ON Canada; ^27^ Rotman Research Institute Baycrest, University of Toronto, Toronto, ON Canada; ^28^ Campbell Family Mental Health Research Institute, Centre for Addiction and Mental Health, Toronto, ON Canada; ^29^ Adult Neurodevelopment and Geriatric Psychiatry Division, CAMH, Toronto, ON Canada; ^30^ Temerty Faculty of Medicine, Division of Psychiatry, University of Toronto, Toronto, ON Canada; ^31^ Baycrest Academy for Research and Education, Toronto, ON Canada; ^32^ Baycrest Hospital, Toronto, ON Canada; ^33^ Robarts Research Institute, University of Western Ontario, London, ON Canada; ^34^ Division of Geriatric Medicine, Western University, London, ON Canada; ^35^ Sunnybrook Research Institute, Toronto, ON Canada; ^36^ Robarts Research Institute, London, ON Canada; ^37^ Parkwood Institute/St Joseph’s Healthcare Center, London, ON Canada; ^38^ Bruyere Continuing Care, Ottawa, ON Canada; ^39^ University of Ottawa, Ottawa, ON Canada; ^40^ University of Calgary, Calgary, AB Canada; ^41^ Hotchkiss Brain Institute, University of Calgary, Calgary, AB Canada; ^42^ Edmond J Safra Program for Parkinson Disease and the Morton and Gloria Shulman Movement Disorder Clinic, Toronto Western Hospital, University Health Network, Toronto, ON Canada; ^43^ Western University, London, ON Canada; ^44^ LC Campbell Cognitive Neurology Research Unit, Sunnybrook Research Institute, Toronto, ON Canada; ^45^ Neurology/Medicine, University of Ottawa, Ottawa, ON Canada; ^46^ Temerty Faculty of Medicine, Department of Laboratory Medicine and Pathobiology, University of Toronto, Toronto, ON Canada; ^47^ LC Campbell Cognitive Neurology Research Unit, Toronto, MA USA; ^48^ The Hospital for Sick Children, Archie’s Cochlear Implant Lab, Toronto, ON Canada; ^49^ Temerty Faculty of Medicine, Department of Psychiatry, University of Toronto, Toronto, ON Canada; ^50^ Keenan Research Centre for Biomedical Science, Li Ka Shing Knowledge Institute, St. Michael's Hospital, Toronto, ON Canada; ^51^ Division of Neurosurgery, Faculty of Medicine, University of Toronto, Toronto, ON Canada

## Abstract

**Background:**

Mild Behavioral Impairment (MBI) is a condition characterized by neuropsychiatric symptoms (NPS) in older adults without dementia, serving as a precursor to various forms of dementia. This study explores the association between NPS and functional connectivity (FC) within the default mode network (DMN), executive control network (ECN), and salience network (SN) across three high‐risk cohorts: mild cognitive impairment (due to Alzheimer’s) (MCI, n = 79), cerebrovascular disease (CVD, n = 144), and Parkinson’s disease (PD, n = 132).

**Method:**

A total of 367 participants were recruited from the Ontario Neurodegenerative Disease Research Initiative (ONDRI). The assessment of NPS utilized the Neuropsychiatric Inventory Questionnaire (NPI‐Q), with symptom severity rated on a scale from 1 to 3 (mild, moderate, severe). Resting‐state FC was analyzed for the DMN, ECN, and SN, using dual regression analysis to generate subject‐specific whole‐brain FC maps for each network. The association between FC maps and NPS scores was examined using FSL’s *randomise* with 5,000 permutations, while controlling for age, sex, and education. Results are presented following cluster False Discovery Rate (FDR) correction.

**Result:**

The study revealed significant associations between NPS and FC specific to each cohort. In the MCI group, disturbed appetite and nighttime behaviors were correlated with increased FC of the dorsal DMN (p<0.05, R = 0.47, and p = 0.01, R = 0.47). The CVD group exhibited correlations between higher levels of anxiety and decreased FC of the dorsal DMN (p<0.05, R = ‐0.4), ventral DMN (p<0.05, R = ‐0.33), and bilateral ECN (p<0.05, R = ‐0.35 and R = ‐0.33). The PD group showed disturbed nighttime behavior associated with increased FC in ventral DMN (p<0.05, R = 0.35) and bilateral ECN (p<0.05, R = 0.43 and R = 0.37).

**Conclusion:**

This research underscores disorder‐specific correlations between specific NPS domains and FC in MCI, CVD, and PD, emphasizing the unique neural underpinnings of symptomatology in each group. Furthermore, it is essential to note the inherent heterogeneity in all groups. Overall, the pathological substrates of neurodegenerative disorders likely play a pivotal role in shaping the neural correlates of MBI within each disorder. These findings provide valuable insights into targeted interventions and avenues for future research in neurodegenerative disorders.

